# Epigenetic Age Acceleration and Disparities in Posttraumatic Stress in Women in Southeast Louisiana: NIMHD Social Epigenomics Program

**DOI:** 10.1001/jamanetworkopen.2024.21884

**Published:** 2024-07-29

**Authors:** Alicia K. Smith, Seyma Katrinli, Dawayland O. Cobb, Evan G. Goff, Michael Simmond, Grace M. Christensen, Tyler Prusisz, Sierra N. Garth, Meghan Brashear, Anke Hüls, Erika J. Wolf, Edward J. Trapido, Ariane L. Rung, Nicole R. Nugent, Edward S. Peters

**Affiliations:** 1 Department of Gynecology and Obstetrics, School of Medicine, Emory University, Atlanta, Georgia; 2 Department of Psychiatry and Behavioral Sciences, School of Medicine, Emory University, Atlanta, Georgia; 3 Department of Human Genetics, School of Medicine, Emory University, Atlanta, Georgia; 4 Department of Epidemiology, Rollins School of Public Health, Emory University, Atlanta, Georgia; 5 Epidemiology Program, Louisiana State University School of Public Health, New Orleans; 6 Epidemiology Department, University of Nebraska Medical Center College of Public Health, Omaha; 7 Gangarosa Department of Environmental Health, Rollins School of Public Health, Emory University, Atlanta, Georgia; 8 Department of Biostatistics and Bioinformatics, Rollins School of Public Health, Emory University, Atlanta, Georgia; 9 National Center for PTSD at VA Boston Healthcare System and Department of Psychiatry, Boston University Chobanian & Avedisian School of Medicine, Boston, Massachusetts; 10 Department of Psychiatry and Human Behavior, Alpert Brown Medical School, Providence, Rhode Island; 11 Department of Pediatrics, Alpert Brown Medical School, Providence, Rhode Island; 12 Department of Emergency Medicine, Alpert Brown Medical School, Providence, Rhode Island

## Abstract

**Importance:**

Disasters experienced by an entire community provide opportunities to understand individual differences in risk for adverse health outcomes over time. DNA methylation (DNAm) differences may help to distinguish individuals at increased risk following large-scale disasters.

**Objective:**

To examine the association of epigenetic age acceleration with probable posttraumatic stress disorder (PTSD) and PTSD symptom severity in women.

**Design, Setting, and Participants:**

This prospective cohort study examined data from participants in the Women and Their Children’s Health cohort, who were characterized longitudinally following the Deepwater Horizon oil spill (DHOS) in 2010 and through numerous hurricanes in the Gulf Coast region of the US. Wave 1 occurred August 6, 2012, through June 26, 2014, and wave 2 occurred September 2, 2014, through May 27, 2016. Data were analyzed between August 18 and November 4, 2023. Address-based sampling was used to recruit women aged 18 to 80 years and residing in 1 of the 7 Louisiana parishes surrounding the DHOS-affected region. Recruitment consisted of 2-stage sampling that (1) undersampled the 2 more urban parishes to maximize probability of participant oil exposure and (2) proportionally recruited participants across census tracts in the 5 other parishes closest to the spill.

**Exposure:**

Posttraumatic stress subsequent to the DHOS.

**Main Outcome and Measures:**

Epigenetic age acceleration was measured by DNAm assayed from survey wave 1 blood samples. Posttraumatic stress disorder was assessed using the PTSD Checklist for *DSM-5* at survey wave 2, and lifetime trauma exposure was assessed using the Life Events Checklist for *DSM-5*. General linear models were used to examine the association between wave 1 DNAm age and wave 2 probable PTSD diagnosis and symptom severity.

**Results:**

A total of 864 women (mean [SD] age, 47.1 [12.0] years; 328 Black [38.0%], 19 American Indian [2.2%], 486 White [56.3%], and 30 of other racial groups, including uknown or unreported [3.5%]) were included. Black and American Indian participants had a higher age acceleration at wave 1 compared with White participants (β = 1.64 [95% CI, 1.02-2.45] and 2.34 [95% CI, 0.33-4.34], respectively), and they had higher PTSD symptom severity at wave 2 (β = 7.10 [95% CI, 4.62-9.58] and 13.08 [95% CI, 4.97-21.18], respectively). Epigenetic age acceleration at wave 1 was associated with PTSD symptom severity at wave 2 after adjusting for race, smoking, body mass index, and household income (β = 0.38; 95% CI, 0.11-0.65).

**Conclusions and Relevance:**

In this cohort study, epigenetic age acceleration was higher in minoritized racial groups and associated with future PTSD diagnosis and severity. These findings support the need for psychoeducation about traumatic responses to increase the likelihood that treatment is sought before years of distress and entrenchment of symptoms and comorbidities occur.

## Key Points

**Question:**

What is the association of epigenetic age acceleration with future development of posttraumatic stress disorder?

**Findings:**

In this cohort study of 864 women in southeast Louisiana, a significantly higher epigenetic age acceleration and faster pace of aging was found among those who would meet criteria for posttraumatic stress disorder within 2 years.

**Meaning:**

These findings suggest that epigenetic age acceleration influences sensitivity to future traumas.

## Teaser

This cohort study examines the association of epigenetic age acceleration with probable posttraumatic stress disorder and symptom severity in US women exposed to disaster.

## Introduction

Environmental and technological disasters are increasingly common,^1,2^ with residents affected by disaster often experiencing more than 1 traumatic experience, making it critically important to understand the impact of trauma burden on psychological and physical health.^3,4,5,6,7,8^ In the US, the Gulf Coast region has faced multiple natural and technological disasters, including the Deepwater Horizon oil spill (DHOS) in 2010 and Hurricanes Katrina and Rita (2005), Gustav (2008), Isaac (2012), and numerous others in 2020 and 2021. Louisiana consistently ranks as one of the worst states for chronic diseases, with poor outcomes observed across cardiovascular health, cancer, asthma, and diabetes.^9^ Disaster is undoubtedly a contributor, with New Orleans showing a more than 3-fold increase in admissions for acute myocardial infarction in the 6 years after Hurricane Katrina compared with 6 years prior and patients with acute myocardial infarction showing more psychiatric comorbidities.^10^ Studies have documented substantial posttraumatic stress disorder (PTSD), depression, and psychological problems following Hurricane Katrina.^4,11,12,13,14^ After the DHOS, residents showed increases in major depression, thoughts of suicide, and suicide plans.^15,16,17,18^ Participants in the Women and Their Children’s Health (WaTCH) cohort, which was characterized longitudinally following the DHOS, reported having higher levels of PTSD symptoms compared with other epidemiologic samples.^19^

Epigenetic changes, such as DNA methylation (DNAm), modify DNA structure to permit molecular adaptability^20,21,22^ and complexity,^23^ with functional changes in DNA products.^24^ Stress and trauma alter DNAm profiles, which then translate to acceleration of cellular aging and premature development of age-related disease and mortality.^25,26^ Prior studies of DNAm have documented associations between trauma and dysregulation of immune and glucocorticoid systems, which may increase vulnerability to diseases of stress and aging.^27,28,29,30,31^ Posttraumatic stress disorder and chronic stress may accelerate cellular aging through oxidative stress, inflammatory processes, autonomic and metabolic processes, and glucocorticoid system activation.^24,30,32,33,34,35^ In particular, PTSD has been associated with measures of accelerated aging.^33,36^ However, most studies have been cross-sectional, and few have evaluated epigenetic age acceleration as a marker of future health problems.

Analytic discoveries permit the use of a weighted collection of DNAm at different locations in the genome, called epigenetic clocks, as a biological measure of aging,^37,38,39^ with epigenetic age acceleration defined as the difference between DNAm-based age and chronologic age. The PhenoAge^40^ clock leverages phenotypic measures of age, such as serum glucose levels and C-reactive protein levels, to capture clinical measures of physical decline along with chronologic age. The GrimAge^41^ clock uses a unique set of DNAm markers trained to capture morbidity and all-cause mortality. Cross-sectional studies of individuals weeks to months post trauma have shown associations between PTSD and GrimAge acceleration,^42,43,44,45^ and GrimAge acceleration at the time of trauma (measured in the emergency department) has been found to be associated with PTSD 6 months later.^36^ A recent investigation comprising veterans with PTSD assessed GrimAge acceleration (as well as markers of neuropathy and inflammation) at baseline and at follow-up approximately 5.5 years later.^46^ Findings suggested that externalizing psychiatric psychopathology, such as antisocial personality and substance abuse symptoms, was associated with accelerated epigenetic aging, which was in turn associated with inflammatory markers. Interestingly, GrimAge acceleration at baseline appeared to precede subsequent increases in biomarkers of neuropathology and inflammation.

Another recently developed clock, DunedinPACE, focuses on the pace of aging.^47^ Unlike approaches developed to predict chronologic age, DunedinPACE capitalizes on longitudinal within-person change over time occurring between 2 blood sample collections to estimate aging processes that affect organ systems over time. DunedinPACE has been shown to have a strong correlation with clinical measures and self-reported health status in adult females.^48^ There is also evidence that the accelerated pace of aging, as measured by the DunedinPACE clock, may be tied to adverse childhood experiences, perceived stress, and a high burden of stressful life events.^49,50^ Furthermore, PTSD measured using both an index of severity and a threshold approach was also associated with an accelerated pace of aging.^50^

This emerging area of research has begun to characterize important processes related to DNAm age, traumatic stress, and PTSD. The multiwave WaTCH cohort, a sample of women aged 18 to 80 years exposed to disaster and high levels of trauma, provides an important opportunity to examine the association between DNAm age markers measured 2 to 4 years following a common disaster exposure (DHOS) with lifetime trauma exposure and PTSD symptoms measured 4 to 6 years after the DHOS. We hypothesized that age acceleration, as an index of poor health overall, at wave 1 of the WaTCH study would be associated with a probable PTSD diagnosis and PTSD symptom severity assessed at wave 2, even after adjusting for commonly observed sociodemographic covariates.

## Methods

This cohort study was approved by the institutional review board of the Louisiana State University Health Sciences Center–New Orleans. All participants provided written informed consent. This report follows the Strengthening the Reporting of Observational Studies in Epidemiology (STROBE) reporting guideline for cross-sectional observational studies.

### Study Design, Setting, and Participants

The WaTCH cohort study was initially undertaken to examine the 2010 DHOS’s short- and long-term health outcomes.^51^ The study focused on women because women represent a vulnerable and influential population that is often central to decision-making processes within families, especially with respect to health, support, diet, and child-rearing. Women participating in the National Institute of Environmental Health Sciences–led Gulf Long-Term Follow-Up Study were not eligible to participate in the WaTCH study.

Methods have been previously described.^51^ Briefly, between August 6, 2012, and June 26, 2014, 2852 women were recruited and enrolled from the 7 most affected parishes (counties) in southeast Louisiana. While the 2 urban parishes were intentionally undersampled because substantial oil spill exposure was unlikely, a proportional representative sample relative to the 2010 census for the other 5 parishes was sought. All enrolled women completed baseline telephone interviews (wave 1); 1233 also participated in an in-person home visit. Blood was obtained from 1058 women who participated in the home visit and consented to a blood draw. Between September 2, 2014, and May 27, 2016, we conducted follow-up interviews (wave 2) of 2038 women. The sample size for this study comprised all women who consented to a blood draw, completed the demographic variable assessment (ie, age and race) in the wave 1 survey, and participated in the trauma exposure and PTSD interviews in wave 2 (eFigure 1 in Supplement 1).

### Independent Variable: Epigenetic Age Acceleration

Blood was collected at wave 1 into EDTA tubes and separated by centrifugation, and buffy coat and plasma were stored separately at −80 °C. DNA was isolated from buffy coats using the QIAamp DNA Mini Kit (Qiagen). DNA methylation was interrogated using the MethylationEPIC, version 2.0 BeadChip (Illumina), and raw methylation β values were determined using the R package minfi. Prior to calculating age acceleration, samples with probe detection call rates of less than 95% and those with a mean intensity value of either less than 50% of the experiment-wide sample mean or less than 2000 arbitrary units were removed using the R package CpGassoc.^52^ Probes with detection *P* > .01 were set to missing. DNA methylation age was calculated using methods described by Levine et al^40^ (PhenoAge) and Lu et al^41^ (GrimAge). Each method estimates age from DNAm of different sets of CpG sites and is trained to reflect age-related health risk and mortality. For each of these epigenetic clocks, epigenetic age acceleration was calculated as the residual between DNAm age and chronologic age. The pace of aging was estimated using the DunedinPACE clock as described by Belsky et al.^47^ The pace of aging estimate provided by the DunedinPACE is scaled to a mean of 1 year of biological aging per year of chronologic aging to facilitate interpretation.

### Dependent Variable: PTSD

Interviews were completed at wave 2 (occurring from 2014 to 2016) to assess trauma exposure and symptoms of PTSD. Women were asked to identify the most distressing event of their lifetime and to report PTSD symptoms experienced over the course of the past month that were associated with the most distressing event using the PTSD Checklist for the *Diagnostic and Statistical Manual of Mental Disorders* (Fifth Edition) (*DSM-5*) (PCL-5).^53^ As described in a previous study in this cohort,^19^ PTSD categorization was calculated by summing the 20 items of the PCL-5, with scores at or above 38 being considered positive for probable PTSD diagnosis.

### Sociodemographic Variables

At wave 1, age, race, household size, marital status, occupation, education, and income were ascertained through self-report during a structured survey interview. Consistent with our prior strategy,^54^ the category of high school graduate included those with a General Educational Development test, vocational training, community college, or some college. Self-reported race response options were consistent with the PhenX Toolkit^55^ and included the following categories: Black, American Indian, White, and other (including unknown or unreported). Due to a small sample size, participants who self-reported Asian or Pacific Islander race were included in the other category. Participants who self-reported more than 1 race were also included in the other category. Race was included in the analysis of health disparities as a potential confounder and effect modifier. For marital status, participants were classified as single if they reported being widowed, divorced, separated, or never married. Women who reported living with a partner were classified as married. Age was calculated from the participants’ birth date to the date of the blood draw visit. Body mass index (BMI), calculated as weight in kilograms divided by height in meters squared, was self-reported and confirmed at the time of the blood draw. We validated the participants’ self-reported smoking status by calculating a DNAm-based smoking score,^56^ which was strongly associated with the self-report (β = 39.4; *R*^2^ = 0.29; *P* < .001). At wave 2, age, household composition, marital status, occupation, and income were ascertained again through self-report. A modified version of the Life Events Checklist for *DSM-5*, a comprehensive assessment of *DSM-5* criterion A traumas including the DHOS, hurricanes, violence exposure, etc, was used to assess trauma event exposure.^57^ This interview permitted characterization of potentially traumatic experiences that participants encountered in addition to their exposure to the DHOS technological disaster.

### Statistical Analysis

The data analysis was performed between August 18 and November 4, 2023. Differences in sociodemographic variables collected at wave 1 and wave 2 (eg, employment status) were tested using χ^2^ tests. Descriptive statistics were calculated for probable PTSD diagnosis, PTSD symptom severity, and sociodemographic variables. Continuous and ordinal variables were assessed using means and SDs, and categorical variables were summarized using numbers and percentages. Differences in sociodemographic variables based on probable PTSD diagnosis were compared using logistic regression models, and differences based on PTSD symptoms were assessed using linear regression models. To test the bivariate association between epigenetic age acceleration and sociodemographic variables, we used linear regression models. For ordinal variables, the largest group was set as the reference group, and pairwise analyses were performed for each group compared with the reference.

To evaluate the multivariable association of probable PTSD diagnosis with epigenetic age acceleration, we identified confounding variables through a directed acyclic graph (eFigure 2 in Supplement 1) using dagitty.net.^58^ The minimally sufficient adjustment set for the direct effect of epigenetic age acceleration on probable PTSD diagnosis was race, tobacco use or smoking, BMI, and income. We performed a series of logistic regression models that controlled for these variables. In model 1, we estimated the unadjusted association between age acceleration at wave 1 and wave 2 and PTSD. We adjusted for race in model 2; for race and smoking in model 3; for race, smoking, and BMI in model 4; and for race, smoking, BMI, and annual household income in model 5. We performed comparable analyses to examine the association between age acceleration and continuous PTSD symptoms, with general linear models including a robust sandwich variance estimate. All hypotheses were considered with 2-sided tests, assuming a significance threshold of *P* < .05. All analyses were conducted using R, version 4.2.1 software (R Foundation for Statistical Computing).

## Results

### WaTCH Cohort Demographic and Behavioral Characteristics

A total of 864 women from the WaTCH study (mean [SD] age, 47.1 [12.0] years) were included in the analysis, with the cohort comprising 328 Black participants (38.0%), 19 American Indian participants (2.2%), 486 White participants (56.3%), and 30 endorsed other racial groups, including unknown or unreported (3.5%) (Table 1). At wave 1, 254 of 862 participants (29.5%) reported having some level of college education, 464 of 861 (53.9%) were employed, and 494 of 834 (59.2%) had an annual household income of less than $50 000. The demographic characteristics reported at wave 1 were consistent with those reported at wave 2. There was no significant change in marital status, household income, or household size. The mean (SD) number of trauma exposures at wave 2 was 6.6 (3.4). In total, 125 participants (14.5%) met the threshold for probable PTSD diagnosis at wave 2.

**Table 1.** Participant Characteristics (N = 864)

Characteristic	No. of participants (%)	
	Wave 1 (August 6, 2012, through June 26, 2014)	Wave 2 (September 2, 2014, through May 27, 2016)
Age, mean (SD), y	47.1 (12.0)	49.2 (12.3)
Race^a^		
American Indian	19 (2.2)	NA
Black	328 (38.0)	NA
White	486 (56.3)	NA
Other^b^	30 (3.5)	NA
Education^a^		
Less than high school	107 (12.4)	NA
High school graduate	501 (58.1)	NA
College or higher	254 (29.5)	NA
Employment^c^		
Unemployed	397 (46.1)	382 (44.4)
Full time	351 (40.8)	362 (42.0)
Part time	113 (13.1)	117 (13.6)
Marital status^c^		
Married or living with partner	521 (60.4)	504 (58.5)
Single	341 (39.6)	357 (41.5)
Household size, mean (SD), members	3.5 (1.5)	3.1 (1.5)
Annual household income, $^c^		
<20 000	223 (26.7)	275 (32.6)
20 001-50 000	271 (32.5)	249 (29.5)
50 001-80 000	162 (19.4)	146 (17.3)
>80 000	178 (21.3)	175 (20.7)
Lifetime smoking status^c^		
No	549 (63.5)	552 (64.0)
Yes	315 (36.5)	311 (36.0)
Probable PTSD diagnosis		
No	NA	739 (85.8)
Yes	NA	125 (14.5)
PTSD symptoms, mean (SD)	NA	16.6 (18.0)
Trauma exposure, mean (SD), events	NA	6.6 (3.4)
BMI, mean (SD)	32.8 (8.8)	NA

Abbreviations: BMI, body mass index (calculated as weight in kilograms divided by height in meters squared); NA, not applicable; PTSD, posttraumatic stress disorder.

^a^ Race and education were only assessed at wave 1. Race was missing for 1 observation, and education was missing for 2 observations.

^b^ The other category included Asian or Pacific Islander, multiracial, do not know, or refused.

^c^ Employment status was missing for 3 participants in both waves. Marital status was missing for 2 participants at wave 1 and 3 at wave 2. For annual household income, 30 participants reported not knowing or refusing to provide at wave 1 and 19 at wave 2. Lifetime smoking status was missing for 1 participant at wave 2.

### Age Acceleration Association With Demographic and Behavioral Characteristics

We noted differences in epigenetic age acceleration among the racial groups for the GrimAge and pace of aging clocks (Table 2), with Black and American Indian participants having higher age acceleration (β = 1.64 [95% CI, 1.02-2.45] and 2.34 [95% CI, 0.33-4.34], respectively) compared with White participants. Lower levels of education were associated with age acceleration across clocks (GrimAge: β = 2.16 [95% CI, 1.26-3.05]; PhenoAge: β = 1.75 [95% CI, 0.19-3.31]; pace of aging: β = 0.05 [95% CI, 0.03-0.08]). Similarly, participants who reported full-time employment had lower GrimAge acceleration (β = −1.55; 95% CI, −2.18 to −0.92) and a slower pace of aging (β = −0.05; 95% CI, −0.06 to −0.03) than those who were unemployed. GrimAge acceleration and pace of aging were also higher in single women compared with those who were married or living with a partner (GrimAge: β = 1.10 [95% CI, 0.49-1.70]; pace of aging: β = 0.04 [95% CI, 0.02-0.06]). Increases in annual household income were associated with significantly less GrimAge acceleration ($20 001-$50 000: β = −1.67 [95% CI, −2.43 to −0.92]; $50 001-$80 000: β = −2.99 [95% CI, −3.86 to −2.13]; >$80 000: β = −3.17 [95% CI, −4.01 to −2.33]) and a slower pace of aging ($20 001-$50 000: β = −0.05 [95% CI, −0.07 to −0.03]; $50 001-$80 000: β = −0.10 [95% CI, −0.12 to −0.07]; >$80 000: β = −0.11 [95% CI, −0.13 to −0.08]). For both clocks, we also noted higher epigenetic age acceleration associated with lifetime smoking (GrimAge: β = 4.05 [95% CI, 3.50-4.60]; pace of aging: β = 0.06 [95% CI, 0.04-0.07]) and higher BMI (GrimAge: β = 0.06 [95% CI, 0.03-0.10]; pace of aging: β = 0.01 [95% CI, 0.00-0.01]). Trauma exposure was not associated with epigenetic age acceleration.

**Table 2.** Bivariate Associations Between Epigenetic Age Acceleration and Cohort Demographic and Behavioral Factors

Characteristic	GrimAge acceleration		PhenoAge acceleration		Pace of aging	
	β (95% CI)	*P* value	β (95% CI)	*P* value	β (95% CI)	*P* value
Race						
American Indian	2.34 (0.33 to 4.34)	.02	3.33 (−0.11 to 6.76)	.06	0.14 (0.08 to 0.20)	<.001
Black	1.64 (1.02 to 2.45)	<.001	0.94 (−0.11 to 1.99)	.08	0.07 (0.06 to 0.09)	<.001
White	1 [Reference]	NA	1 [Reference]	NA	1 [Reference]	NA
Other^a^	1.25 (−0.36 to 2.86)	.13	−0.30 (−3.07 to 2.46)	.83	0.04 (−0.01 to 0.09)	.10
Education						
Less than high school	2.16 (1.26 to 3.05)	<.001	1.75 (0.19 to 3.31)	.03	0.05 (0.03 to 0.08)	<.001
High school graduate	1 [Reference]	NA	1 [Reference]	NA	1 [Reference]	NA
College or higher	−1.69 (−2.33 to 1.04)	<.001	0.14 (−0.99 to 1.27)	.81	−0.05 (−0.07 to −0.03)	<.001
Employment						
Unemployed	1 [Reference]	NA	1 [Reference]	NA	1 [Reference]	NA
Full time	−1.55 (−2.18 to −0.92)	<.001	−0.82 (−2.00 to 0.26)	.14	−0.05 (−0.06 to −0.03)	<.001
Part time	−0.47 (−1.38 to 0.45)	.31	0.20 (−1.37 to 1.77)	.80	−0.01 (−0.03 to 0.02)	.72
Marital status						
Married or living with partner	1 [Reference]	NA	1 [Reference]	NA	1 [Reference]	NA
Single	1.10 (0.49 to 1.70)	<.001	0.49 (−0.54 to 1.51)	.35	0.04 (0.02 to 0.06)	<.001
Household income, $						
<20 000	1 [Reference]	NA	1 [Reference]	NA	1 [Reference]	NA
20 001-50 000	−1.67 (−2.43 to −0.92)	<.001	−0.01 (−1.33 to 1.32)	.99	−0.05 (−0.07 to −0.03)	<.001
50 001-80 000	−2.99 (−3.86 to −2.13)	<.001	−0.87 (−2.39 to 0.64)	.26	−0.10 (−0.12 to −0.07)	<.001
>80 000	−3.17 (−4.01 to −2.33)	<.001	−0.73 (−2.20 to 0.74)	.33	−0.11 (−0.13 to −0.08)	<.001
Lifetime smoking status						
No	1 [Reference]	NA	1 [Reference]	NA	1 [Reference]	NA
Yes	4.05 (3.50 to 4.60)	<.001	1.06 (0.02 to 2.10)	.045	0.06 (0.04 to 0.07)	<.001
Trauma exposure	0.05 (−0.04 to 0.14)	.24	0.11 (−0.03 to 0.26)	.13	0.00 (−0.00 to 0.01)	.08
BMI	0.06 (0.03 to 0.10)	<.001	0.12 (0.06 to 0.17)	<.001	0.01 (0.00 to 0.01)	<.001

Abbreviations: BMI, body mass index; NA, not applicable.

^a^ The other category included Asian or Pacific Islander, multiracial, do not know, or refused.

### Age Acceleration Association With PTSD

We noted differences in some sociodemographic characteristics for participants who met criteria for probable PTSD diagnosis and PTSD symptom severity at wave 2 (Table 3). Compared with White participants, more Black and American Indian participants met the criteria for probable PTSD (odds ratio [OR], 2.21 [95% CI, 1.48-3.33] and 5.32 [95% CI, 1.90-13.90], respectively) and had higher PTSD symptom severity (β = 7.10 [95% CI, 4.62-9.58] and 13.08 [95% CI, 4.97-21.18], respectively). Lower education was associated with probable PTSD (OR, 2.63; 95% CI, 1.61-4.24) and higher PTSD symptom severity (β = 8.49; 95% CI, 4.80-12.18). Full-time employment was associated with a lower likelihood of probable PTSD (OR, 0.40; 95% CI, 0.25-0.61) and less PTSD symptom severity (β = −7.69; 95% CI, −10.2 to −5.14). Being single was associated with both probable PTSD (OR, 1.92; 95% CI, 1.31-2.82) and higher PTSD symptom severity (β = 6.22; 95% CI, 3.80-8.64). Participants with probable PTSD were less likely to have annual household incomes above $50 000 ($50 001-$80 000: OR, 0.21 [95% CI, 0.09-0.42]; >$80 000: OR, 0.22 [95% CI, 0.10-0.42]), with PTSD symptom severity inversely associated with household income ($50 001-$80 000: β = −11.37 [95% CI, −14.82 to −7.92]; >$80 000: β = −14.22 [95% CI, −17.48 to −10.97]). There was no significant difference in age, household size, smoking history, or BMI between participants with probable PTSD and control participants exposed to trauma. However, higher BMI was associated with higher PTSD symptom severity (β = 0.19; 95% CI, 0.05-0.32).

**Table 3.** Demographic and Behavioral Differences Associated With Probable PTSD Diagnosis at Wave 2

Characteristic	Probable PTSD diagnosis				Total PTSD symptom severity	
	No. of participants (%)^a^		OR (95% CI)	*P* value^b^	β (95% CI)	*P* value^b^
	Control (n = 739)	PTSD (n = 125)				
Age, mean (SD), y	49.5 (12.5)	47.3 (11.4)	0.99 (0.97 to 1.00)	.07	−0.08 (−0.18 to 0.01)	.09
Race						
American Indian	12 (1.6)	7 (5.6)	5.32 (1.90 to 13.90)	<.001	13.08 (4.97 to 21.18)	.002
Black	264 (35.7)	64 (51.2)	2.21 (1.48 to 3.33)	<.001	7.10 (4.62 to 9.58)	<.001
White	438 (59.3)	48 (38.4)	1 [Reference]	NA	1 [Reference]	NA
Other^c^	25 (3.4)	5 (4.0)	1.82 (0.59 to 4.63)	.24	6.32 (−0.20 to 12.84)	.06
Education						
Less than high school	75 (10.1)	32 (25.6)	2.63 (1.61 to 4.24)	<.001	8.49 (4.80 to 12.18)	<.001
High school graduate	431 (58.3)	70 (56.0)	1 [Reference]	NA	1 [Reference]	NA
College or higher	231 (31.3)	23 (18.4)	0.61 (0.37 to 0.99)	.054	−4.46 (−7.13 to −1.80)	.001
Employment						
Unemployed	307 (41.5)	75 (60.0)	1 [Reference]	NA	1 [Reference]	NA
Full time	330 (44.7)	32 (25.6)	0.40 (0.25 to 0.61)	<.001	−7.69 (−10.24 to −5.14)	<.001
Part time	99 (13.4)	18 (14.4)	0.74 (0.41 to 1.28)	.30	−2.16 (−5.83 to 1.51)	.25
Marital status						
Married or living with partner	448 (60.6)	56 (44.8)	1 [Reference]	NA	1 [Reference]	NA
Single	288 (39.0)	69 (55.2)	1.92 (1.31 to 2.82)	<.001	6.22 (3.80 to 8.64)	<.001
Household size, mean (SD), members	3.1 (1.5)	3.1 (1.5)	1.01 (0.89 to 1.15)	.87	0.19 (−0.63 to 1.01)	.65
Household income, $						
<20 000	215 (29.1)	60 (48.0)	1 [Reference]	NA	1 [Reference]	NA
20 001-50 000	204 (27.6)	45 (36.0)	0.79 (0.51 to 1.21)	.29	−4.69 (−7.64 to −1.75)	.002
50 001-80 000	138 (18.7)	8 (6.4)	0.21 (0.09 to 0.42)	<.001	−11.37 (−14.82 to −7.92)	<.001
>80 001	165 (22.3)	10 (8.0)	0.22 (0.10 to 0.42)	<.001	−14.22 (−17.48 to −10.97)	<.001
Lifetime smoking status						
No	479 (65.0)	73 (58.5)	1 [Reference]	NA	1 [Reference]	NA
Yes	259 (35.0)	52 (41.5)	1.32 (0.89 to 1.93)	.16	2.45 (−0.05 to 4.96)	.055
Trauma exposure, mean (SD), events	6.3 (3.2)	8.0 (4.0)	1.14 (1.08 to 1.20)	<.001	1.37 (1.03 to 1.71)	<.001
BMI, mean (SD)	32.6 (8.7)	33.9 (9.5)	1.02 (0.99 to 1.04)	.13	0.19 (0.05 to 0.32)	.007

Abbreviations: BMI, body mass index (calculated as weight in kilograms divided by height in meters squared); NA, not applicable; OR, odds ratio; PTSD, posttraumatic stress disorder.

^a^ Missing observations were 1 for race, 2 for education, 3 for employment, 3 for marital status, 19 for household income, and 1 for lifetime smoking status.

^b^ The *P* values were calculated from bivariate logistic regressions for comparing the control and probable PTSD groups.

^c^ The other category included Asian or Pacific Islander, multiracial, do not know, or refused.

In unadjusted models (model 1), participants with higher wave 1 age acceleration and a faster pace of aging were more likely to have probable PTSD and higher PTSD symptom severity at wave 2 (Table 4). As more covariates were added to the GrimAge acceleration analyses, the association with probable PTSD was attenuated, though GrimAge acceleration remained associated with PTSD symptoms (in model 5 [adjusted for race, smoking, BMI, and income]: β = 0.38; 95% CI, 0.11-0.65). PhenoAge acceleration was associated with probable PTSD diagnoses in all models, while its association with PTSD symptoms were attenuated as more covariates were added until the association became nonsignificant in model 5 (β = 0.03; 95% CI, 0.00-0.06). Finally, the pace of aging did not remain associated with probable PTSD or PTSD symptoms after controlling for all covariates.

**Table 4.** Multivariable Association of Age Acceleration and Both Probable PTSD Diagnosis and Symptom Severity

Characteristic and model^b^	GrimAge acceleration^a^		PhenoAge acceleration		Pace of aging	
	β (95% CI)	*P* value	β (95% CI)	*P* value	β (95% CI)	*P* value
Probable PTSD diagnosis						
1	0.07 (0.03 to 0.10)	<.001	0.04 (0.01 to 0.06)	.004	2.43 (1.04 to 3.82)	<.001
2	0.06 (0.01 to 0.10)	.009	0.03 (0.01 to 0.06)	.01	1.71 (0.25 to 3.18)	.02
3	0.06 (0.01 to 0.10)	.009	0.03 (0.01 to 0.06)	.02	1.34 (−0.19 to 2.86)	.08
4	0.06 (0.01 to 0.10)	.009	0.03 (0.00 to 0.06)	.02	1.24 (−0.37 to 2.85)	.13
5	0.04 (0.00 to 0.09)	.06	0.03 (0.00 to 0.06)	.02	0.53 (−1.11 to 2.19)	.52
PTSD symptoms						
1	0.69 (0.39 to 0.98)	<.001	0.24 (0.08 to 0.41)	<.001	24.68 (16.0 to 33.30)	<.001
2	0.56 (0.29 to 0.83)	<.001	0.21 (0.03 to 0.35)	.008	18.43 (9.46 to 27.40)	<.001
3	0.56 (0.29 to 0.83)	<.001	0.19 (0.03 to 0.35)	.02	15.67 (6.40 to 24.90)	<.001
4	0.55 (0.28 to 0.82)	<.001	0.17 (0.01 to 0.33)	.03	14.24 (4.35 to 24.12)	.005
5	0.38 (0.11 to 0.65)	.006	0.15 (0.00 to 0.31)	.06	7.66 (−2.24 to 17.66)	.13

Abbreviation: PTSD, posttraumatic stress disorder.

^a^ GrimAge incorporates a methylation-based estimate of smoking pack-years. Therefore, self-reported smoking is not controlled for in this clock’s models.

^b^ Model 1 was estimated with no covariates. Model 2 controls for race. Model 3 controls for race and smoking. Model 4 controls for race, smoking, and body mass index. Model 5 controls for race, smoking, body mass index, and income.

## Discussion

The goal of this cohort study was to examine the hypothesis that epigenetic age acceleration is longitudinally associated with probable PTSD diagnosis and PTSD symptom severity years later. The hypothesis was examined in women from the WaTCH cohort who had blood drawn at wave 1 of the study and PTSD symptoms assessed at wave 2. Our bivariate analysis showed that epigenetic age acceleration was more pronounced in Black and American Indian participants, as well as in those who reported lower levels of education and lower income. Epigenetic age acceleration was also more likely in participants who reported being unemployed vs working full time, those who smoked, and those with a higher BMI. Age acceleration evaluated using the GrimAge and PhenoAge epigenetic clocks, which were designed to quantify age-related progress at the point of sampling, showed higher epigenetic age acceleration at wave 1 among participants who would meet criteria for probable PTSD and who had higher PTSD symptom severity at wave 2, though the strength of the associations was attenuated to varying degrees when controlling for race, smoking, BMI, and income. When we examined the DunedinPACE clock, which was designed to estimate the prospective rate of age-related decline, we found a faster pace of aging at wave 1 in participants who would meet criteria for probable PTSD and who had higher PTSD symptoms at wave 2, though the results were attenuated rapidly as covariates were added to the model. Collectively, these data suggest that overall poorer health at wave 1 was a risk factor for future PTSD and that these measures of age-related physical decline may be useful for identifying future psychiatric risk.

The present research extends past work that generally assessed DNAm and PTSD contemporaneously with assumptions that PTSD precedes or drives the DNAm age acceleration.^42,44^ We found that in a sample of women exposed to disaster, DNAm alterations consistent with accelerated aging were associated with PTSD symptoms assessed years later. All participants in our sample were recruited from a defined geographic region affected by the DHOS, reported a range of traumas and exposures, and were assessed longitudinally. As our group has reported previously,^19^ participants in the present study reported particularly high levels of trauma exposure, with the count of traumas (mean [SD], 6.6 [3.4]) endorsed at roughly double the levels observed in most epidemiologic samples. This high burden of trauma is contrasted by US epidemiologic research supporting a mean of 3.30 and a mode of 3 lifetime exposures meeting *DSM-5* criteria.^59^ Recent research examining pace of aging found that pace was fastest among individuals with PTSD, followed to a lesser degree by individuals who did not have PTSD but had trauma exposure; individuals who reported no trauma (and thus no PTSD) had the slowest pace of aging.^50^ It is difficult to know why the degree of trauma exposure was not associated with epigenetic age acceleration in our study. It is possible that trauma exposure, which was higher than in most samples,^59^ was sufficiently high to involve ceiling effects. A ceiling effect may be especially true in this Gulf Coast sample of women given their high levels of disaster exposure.

Importantly, our study shows that probable PTSD diagnosis and its symptom severity at wave 2 was not evenly distributed across the cohort. Participants with probable PTSD were more likely to report their race as Black, American Indian, or multiracial. They also had lower levels of education, employment, and household income and were more likely to be single. Education, employment, and household income are important social determinants of health (SDOH), each of which was independently associated with epigenetic age acceleration. Each of these characteristics was associated with race, with participants in a minoritized racial group having lower levels of each determinant. Although we did not have measures of racial discrimination in this study, our observations show that those in minoritized racial groups had higher epigenetic age acceleration, suggesting that there is a cumulative consequence of these SDOH that may increase the risk for PTSD, age-related morbidities, or even early mortality. Recent research in a population-based sample of 470 socioeconomically diverse men and women residing in Baltimore, Maryland, reported that a faster pace of aging was associated with SDOH, including household income below the poverty level and Black race.^60^

Racial and ethnic disparities in accelerated aging and associated psychological and physical health concerns are consistent with the “weathering” hypothesis proposed to understand poorer health observed among Black and minoritized women.^61^ The weathering hypothesis, arising from research characterizing maternal patterns of neonatal mortality in Black compared with White women, proposes that the cumulative effects of socioeconomic disadvantage may result in acceleration of age-related physical health concerns in Black women. Exposure to increased neighborhood violence, and a host of associated symptoms, may be an important contributor to these disparities.^62,63,64,65,66,67^ Studies have increasingly characterized how experiences of discrimination also directly contribute to the weathering process for Black women in the US.^67,68,69^

### Limitations

Although our study provides important insight into epigenetic age acceleration in a community of women with a high trauma exposure who have experienced technological disaster, there are some limitations. First, because PTSD was not assessed at wave 1, it was not possible to examine the ways that preexisting PTSD may have been associated with age acceleration at wave 1. Additional research is needed to explore the potential bidirectional associations between PTSD and age-related processes. It was also not possible to adjust for trauma exposure at wave 1, though this concern is mitigated by the fact that the Life Events Checklist for *DSM-5* is a cumulative and lifetime measure of trauma exposure and was not associated with epigenetic age acceleration. Second, the study was conducted entirely in women, and the results may not generalize to men. Third, given the health disparities observed in these analyses, an important limitation of this study is that we did not explore participant reports of experienced discrimination. Furthermore, it would have been ideal to have an even larger sample of individuals who are members of minoritized communities and to have assessed participants’ experiences with discrimination and racism. Research is under way of a third wave of data collection with a second blood sample collection. These wave 3 assessments include trauma exposure, PTSD, and measures of participant-reported experiences of racism and discrimination.

## Conclusions

The results of this cohort study provide important information about the prospective value of accelerated epigenetic aging in a sample of women who have all experienced a technological disaster and who, as a group, have substantial trauma exposure. As described in previous research examining trauma in the WaTCH cohort,^19^ nearly all participants experienced (often multiple) traumas, including numerous natural disasters and high levels of physical and sexual assault. It is important to note that following a trauma, women are more than twice as likely to develop PTSD as men,^70,71,72,73^ and epidemiologic studies have consistently shown that the prevalence of PTSD is higher among women.^74^ Our findings highlight the association between accelerated aging and PTSD and underscore the critical need for awareness of PTSD symptoms, particularly in areas where disasters are common, so that women recognize their symptoms and seek effective treatment. Both natural and technological disasters may become catalysts for education about the symptoms of and availability of effective treatments for PTSD. Future public health interventions ideally could provide information regarding a host of psychological and physical health outcomes of trauma, ensuring that survivors understand that disasters may indeed be considered traumatic. For some individuals, especially in regions where disasters are more common and where neighbors and friends do not seem to view disasters as traumatic, psychoeducation about traumatic responses may go a long way toward increasing the likelihood that treatment is sought before years of distress and entrenchment of symptoms and comorbidities occur. In addition, for women who may be experiencing PTSD symptoms and comorbid concerns related to other traumatic experiences, such as sexual assault, the ability to approach treatment in the context of their experience with a disaster offers a pathway to treatment and formation of trust with a treatment professional.

## Notes

Supplement 1. **eFigure 1.** Study Flow Diagram **eFigure 2.** Directed Acyclic Graph (DAG) Depicting the Associations Between the Epigenetic Age Acceleration (EAA) and PTSD

Supplement 2.
Data Sharing Statement
