# Supplementary material for: Epigenetic Age Acceleration and Disparities in Posttraumatic Stress in Women in Southeast Louisiana: NIMHD Social Epigenomics Program
Source: JAMA Netw Open. 2024 Jul 29;7(7):e2421884. doi: 10.1001/jamanetworkopen.2024.21884 (PMC11287391; doi:10.1001/jamanetworkopen.2024.21884)
Supplement: Supplement 1. — eFigure 1. Study Flow Diagram eFigure 2. Directed Acyclic Graph (DAG) Depicting the Associations Between the Epigenetic Age Acceleration (EAA) and PTSD [file jamanetwopen-e2421884-s001.pdf]

Supplemental Online Content

Smith AK, Katrinli S, Cobb DO, et al. Epigeneitc age acceleration and disparities in posttraumatic stress in women in southeast Louisiana: NIMHD Social Epigenomics Program. *JAMA Netw Open*. 2024;7(7.2):e2421884. doi:10.1001/jamanetworkopen.2024.21884

eFigure 1. Study Flow Diagram

eFigure 2. Directed Acyclic Graph (DAG) Depicting the Associations Between the Epigenetic Age Acceleration (EAA) and PTSD

This supplemental material has been provided by the authors to give readers additional information about their work.

© 2024 Smith AK et al. *JAMA Network Open*.

eFigure 1. Study Flow Diagram

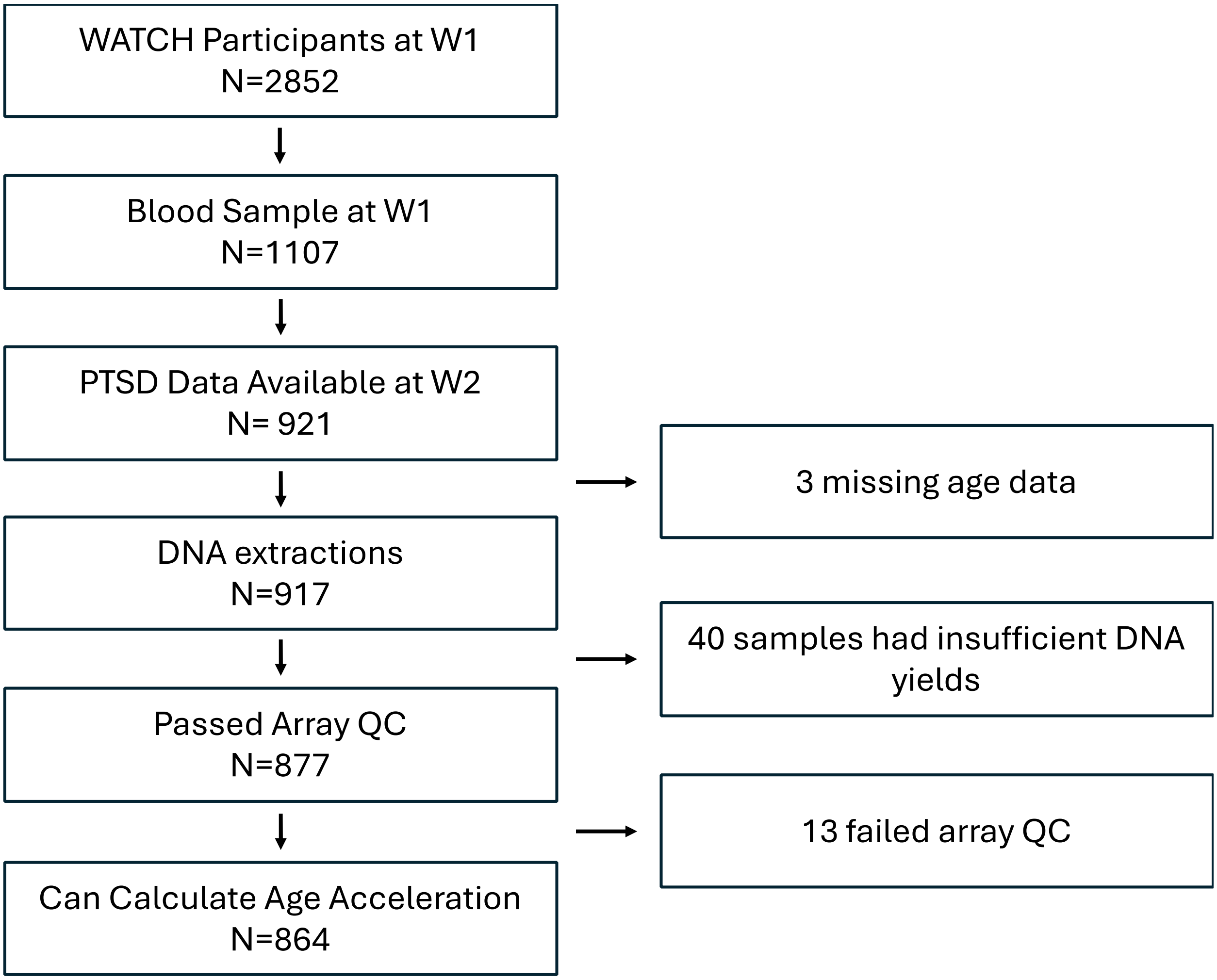

© 2024 Smith AK et al. *JAMA Network Open*.

eFigure 2. Directed Acyclic Graph (DAG) Depicting the Associations Between the Epigenetic Age Acceleration (EAA) and PTSD. Green lines indicate causal or mediating paths. Pink lines indicate confounding paths. The minimally sufficient adjustment set (MSAS) for direct effect of EAA on PTSD = Race + Tobacco Use + BMI + Income. Generated with daggity.net.

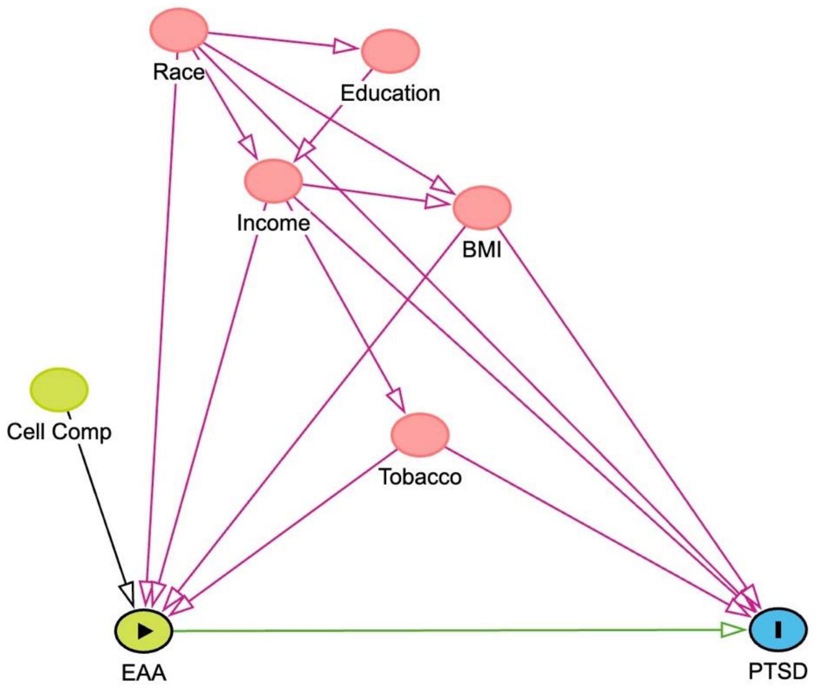

© 2024 Smith AK et al. *JAMA Network Open*.
