# Supplementary material for: Epigenetic Age Acceleration and Disparities in Posttraumatic Stress in Women in Southeast Louisiana: NIMHD Social Epigenomics Program
Source: JAMA Netw Open. 2024 Jul 29;7(7):e2421884. doi: 10.1001/jamanetworkopen.2024.21884 (PMC11287391; doi:10.1001/jamanetworkopen.2024.21884)
Supplement: Supplement 2. — Data Sharing Statement [file jamanetwopen-e2421884-s002.pdf]

Data Sharing Statement

Smith. Epigenetic Age Acceleration and Disparities in Posttraumatic Stress in Women in Southeast Louisiana. *JAMA Netw Open*. Published July 29, 2024.
doi:10.1001/jamanetworkopen.2024.21884

Data
Data available: Yes
Data types: Deidentified participant data, Data dictionary
How to access data: For access to the WaTCH data, including the data used in this paper, contact the study PI, Dr. Edward Peters at epeters@unmc.edu.
When available: With publication

Supporting Documents
Document types: None

Additional Information
Who can access the data: Researchers whose proposed use of the data has been approved by the study PI.
Types of analyses: Approved secondary analyses.
Mechanisms of data availability: Data will be made available after approval of a proposal, which may include a collaboration agreement.
